# The Development of Biomimetic Aligned Skeletal Muscles in a Fully 3D Printed Microfluidic Device

**DOI:** 10.3390/biomimetics7010002

**Published:** 2021-12-21

**Authors:** Rodi Abdalkader, Satoshi Konishi, Takuya Fujita

**Affiliations:** 1 Ritsumeikan Global-Innovation Research Organization (R-GIRO), Ristumeikan University, Kyoto 525-8577, Japan; konishi@se.ritsumei.ac.jp (S.K.); fujita-t@ph.ritsumei.ac.jp (T.F.); 2 Department of Mechanical Engineering, Ritsumeikan University, Kyoto 525-8577, Japan; 3 Department of Pharmaceutical Sciences, Ritsumeikan University, Kyoto 525-8577, Japan

**Keywords:** skeletal muscles, micropattern, fused deposition modeling (FDM)-3D printer

## Abstract

Human skeletal muscles are characterized by a unique aligned microstructure of myotubes which is important for their function as well as for their homeostasis. Thus, the recapitulation of the aligned microstructure of skeletal muscles is crucial for the construction of an advanced biomimetic model aimed at drug development applications. Here, we have developed a 3D printed micropatterned microfluid device (3D-PMMD) through the employment of a fused deposition modeling (FDM)-based 3D printer and clear filaments made of biocompatible polyethylene terephthalate glycol (PETG). We could fabricate micropatterns through the adjustment of the printing deposition heights of PETG filaments, leading to the generation of aligned half-cylinder-shaped micropatterns in a dimension range from 100 µm to 400 µm in width and from 60 µm to 150 µm in height, respectively. Moreover, we could grow and expand C2C12 mouse myoblast cells on 3D-PMMD where cells could differentiate into aligned bundles of myotubes with respect to the dimension of each micropattern. Furthermore, our platform was applicable with the electrical pulses stimulus (EPS) modality where we noticed an improvement in myotubes maturation under the EPS conditions, indicating the potential use of the 3D-PMMD for biological experiments as well as for myogenic drug development applications in the future.

## 1. Introduction

In recent years, there has been a drastic increase in neuromuscular diseases worldwide, necessitating the development of novel therapeutics drugs characterized by high efficacy and safety [1,2]. However, in the early process of drug development, false prediction of results is a problematic matter that normally results in a huge waste of R and D costs and time [3]. Therefore, there is an urgent need to develop an in vitro model that can mimic both the anatomical structure and the physiological functions of human skeletal muscles.

The conventional models of skeletal muscles are based on growing myoblast cells in two-dimensional (2D) culturing dishes followed by an extension culturing period for obtaining myotubes [4]. However, this model lacks the biomimetic three-dimensional (3D) structure of the human tissue [5]. This is particularly true given that skeletal myotubes are characterized by a unique topography represented in their aligned architecture that also imposes a great impact on their function [6]. More recently, 3D models of the skeletal muscles have also been developed through the employment of extracellular matrix (ECM) gels that could provide the mechanical cues for cells to expand and differentiate into myotubes [7,8,9]. However, it is difficult to recapitulate the dynamic conditions in these models including the recapitulation of blood flow and other factors such as mechanical stimuli [10].

The emergence of organ on chip (OOC), also known as micro physiological models, has boosted the development of dynamic biomimetic in vitro models of different human organs such as the intestines [11,12], liver [13], lung [14], eye [15,16], and others [17,18]. There has been a special interest in the development of a micropatterned OOC designed for growing skeletal muscles using microfabrication techniques such as photo lithography [19], soft lithography [20], film deposition [21], electron beam lithography [22], molecular self-assembly [23], and electrically induced patterning [24]. However, these methods encounter sophisticated fabrication processes that demand specific expertise, the use of expensive raw materials, as well as the employment of different bio-adhesive or bio-incompatible materials that can affect cells’ viability and growth.

In fact, in OOC development, the initial prototyping of devices is essential to construct a robust micropatterned OOC while experimenting with different biocompatible materials as well as the optimization of device dimensions and topography of the micropattern. However, performing this using the microfabrication methods requires expertise, time, and additional costs.

Recently there has been a huge development in 3D printing technology in the biomedical field. In the OOC-related field, laser photo lithography-based 3D printers are mainly used for the fabrication of molds designed for shaping casting materials such as polydimethylsiloxane (PDMS), which is utilized in cell culturing [15,25]. However, it is difficult to utilize entirely 3D printed devices made with the photo lithography-based 3D printer due to the utilization of toxic resins as well as the employment of organic solvent in the process of cleaning which can be problematic for cell culturing [26]. In contrast, fused deposition modeling (FDM)-based 3D printer counts on the use of filaments made of biocompatible polymers such as polylactic acid (PLA) and polyethylene terephthalate glycol (PETG) that are commonly used in medical equipment [27,28,29]. This printing technology has already been employed in the fabrication of biomimetic scaffolds of the human bone, heart, and skin using biocompatible polymers [30]. Moreover, as FDM depends on printing a single layer by a layer of polymers [31], it is possible to create micropatterns for the construction of aligned skeletal muscles by controlling the printing deposition heights, the extruder diameters, and the speed of the printing process.

Herein, we have employed FDM-based 3D printing technology for the fabrication of micropatterned microfluidic device made of a PETG clear polymer referred to as 3D-PMMD for the growing and the expansion of skeletal myotubes. We have investigated the adherence of cells as well as their orientation on various aligned micropatterned devices as compared with cells that were grown on conventional dishes with flat surfaces. Finally, we evaluated the integration of the micropatterned devices with the electrical stimulus modality for the assessment of the maturation of skeletal myotubes under normal conditions as well as under electrical stimuli conditions.

## 2. Materials and Methods

### 2.1. Microfluidic Device Fabrication and Characterization

The 3D-PMMD was fabricated by using FDM 3D-printing techniques (da Vinci Jr. ProX, XYZprinting Co., Ltd., Bangkok, Thailand) provided with 1.75 mm PETG filaments (XYZprinting, Inc). The designs of the device were issued by OpenSCAD where each device contained four chambers aimed for culturing cells (cell chamber dimensions: 15 mm length, 2.2 mm width, and 1.8 mm height). Then STL files of the designs were sliced and processed using XYZ print V. 2.0.3 (XYZ printing Co., Ltd., Bangkok, Thailand) considering the following parameters: layers height: 0.1 mm, 0.2 mm, 0.3 mm, or 0.4 mm; shell thickness: 2–10 layers.

For the fabrication of PDMS stamps, Sylgard 184 PDMS two-part elastomer (ratio of pre-polymer to curing agent, 10:1; Dow Corning Corporation, Midland, MI, USA) was mixed, poured into the molds made with different deposition heights of PETG filaments, respectively, and degassed. The PDMS molds were then cured in an oven at 65 °C for 6 h. After curing, the PDMS was removed from the molds, trimmed, and cross-sections of 1 mm were made with a blade. Scanning electron microscopy (SEM) analysis of the micropattern in the devices was performed using KEYENCE (VE-9800, Tokyo, Japan).

### 2.2. C2C12 Culturing in 3D-PMMD

C2C12 myoblast cells were provided by RIKEN Bioresource Research Centre (Ibaraki, Japan). Cells were cultured in DMEM supplemented with 10% (*v*/*v*) fetal bovine serum. The cells were passaged with trypsin-EDTA (0.25–0.02%) solution at a 1:10 subculture ratio. To induce cells differentiation into myotubes, the culturing medium was changed into DMEM supplemented with 2% (*v*/*v*) horse serum.

For seeding cells into 3D-PMMD, microfluidic devices were placed under ultraviolet light in a biosafety cabinet for 30 min. Then microfluidic channels were washed with PBS two times. After that, cell channels were coated with collagen IV from calfskin (Sigma-C9791) in a concentration of 1 mg/mL for 60 min at 37 °C. Cells were harvested using trypsin and collected in a 15 mL tube. Following centrifugation, the cells were resuspended in DMEM medium and introduced into the cells chambers via the cell inlet at a density of 1 × 10^6^ cells/mL. Microfluidic devices were then placed in a humidified incubator at 37 °C with 5% CO_2_ for 24 hrs. Then the culturing medium was replaced with DMEM supplemented with 2% (*v*/*v*) horse serum for four to five days. The medium in each chamber was periodically changed every 24 h.

### 2.3. Electrical Stimulus, Immunofluorescence, and Microscopy Analysis

For the electrical stimulus test, carbon electrodes in diameter of Φ2.0mm were employed. The probes were fixed in the inlets and outlets ports of 3D-PMMD and then connected to an electricity generator (FGX-295, Texio) while controlled by a PC using LabVIEW 2017 software (National Instruments Corporation). Parameters were adjusted to a pulse of 2 ms, intensity of 5 V or 10 V, and frequency of 1Hz. The electrical pulse stimulus (EPS) was adjusted to one stimulus per second.

For immunostaining, cells were fixed with 4% paraformaldehyde in PBS for 25 min at 25 °C and then permeabilized with 0.5% Triton X-100 in PBS for 10 min at 25 °C. Subsequently, the cells were blocked with blocking buffer [5% bovine serum albumin, 0.1% (*v/v*) Tween-20] at room temperature for 90 min and then incubated at 4 °C overnight with the primary antibodies in blocking buffer (Alexa Fluor 594 anti-MYH, 1:100; Santa Cruz, sc-376157 AF594). Cells were then incubated with 4,6-diamidino-2-phenylindole (DAPI) or anti-phalloidin for F-actin using ActinGreenTM 488 (Invitrogen-2277811) at 25 °C for 1 h. For imaging, we used a fluorescence microscope (KEYENCE, Tokyo, Japan) for the acquirement of cell images from cell chambers. Cell orientation was analyzed using ImageJ software (National Institute of Health, Maryland, USA) using the OrientationJ plugin, while Cell Profiler software (V. 3.1.8; Broad Institute of Harvard and MIT, USA) was used for the determination of cells morphology and for the determination of the percentage of MYH positive cells by using pipeline provided three-step analysis: (1) the identification of the primary nucleus objects by using the auto or the Otsu segmentation of the primary nucleus based on their diameter and the fluorescence intensity of the nucleus stain DAPI; (2) the identification of the secondary cells body objects by using fluorescence localization in the cell body; and (3) the determination of the morphological descriptors such as area [32].

### 2.4. Statistical Analysis and Data Visualization

The unpaired t-test and Tukey’s HSD comparison test were performed on samples populations of triplicates or more using Python Jupyter notebook 6.1.4 with Pandas and Bioinfokit packages [33]. Data visualization was performed by Python 3 using matplotlib and seaborn packages.

## 3. Results

### 3.1. Design, Fabrication and Characteristics of 3D-PMMD

**Figure 1:**
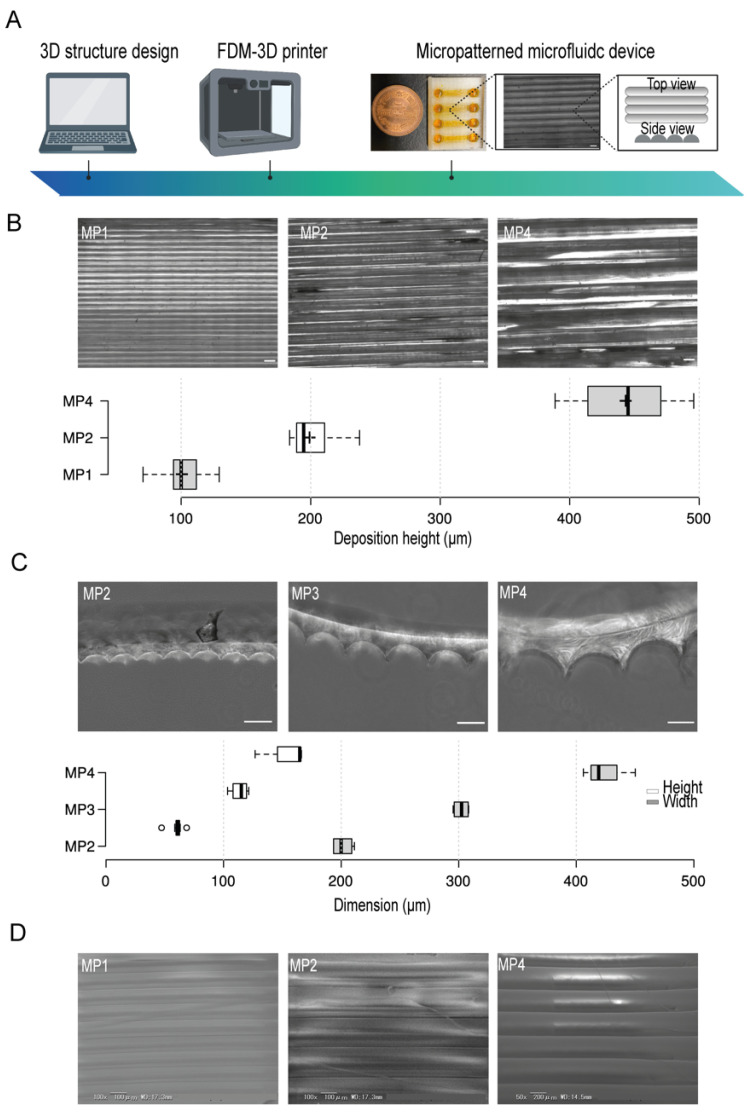
The conceptual design, fabrication, and micropatterns characteristics in the microfluidic devices. (**A**) An illustration of the stepwise design and fabrication of 3D-PMMD showing the micropattern topography. (**B**) (Top) Bright-field images of micropatterns at different printing deposition heights of 100 µm (MP1), 200 µm (MP2), and 400 µm (MP4), (Bottom) Box plot showing the actual layers deposition height measurements in the microfluidic post-3D printing; box limits indicate the 25th and 75th percentiles; Scale bar of 200 µm. (**C**) (Top) Bright-field images of the PDMS stamps cross-section of micropatterns at different printing deposition heights of 200 µm (MP2), 300 µm (MP3), and 400 µm (MP4), (Bottom) Box plot showing the heights and widths of the micropatterns according to the PDMS stamps; box limits indicate the 25th and 75th percentiles. (**D**) Scanning electron microscopy (SEM) images of micropatterns at different printing deposition heights of 100 µm (MP1), 200 µm (MP2), and 400 µm (MP4). Illustration icons were created with BioRender.com under the academic licensing right in s (access on 11 October 2021).

OpenScad software was used for the design of the microfluidic device. A clear type of PETG filament was employed and micropattern aligned lines were generated by the control of the filament’s deposition layers thickness during printing (Figure 1A). To test the resolution of the micropatterns, we first prepared several micropatterns at different printing deposition heights of 100 µm, 200 µm, and 400 µm (Figure 1B) and then investigated their actual dimensions by using bright field microscopy which indicated a correlation between the experimental printing results and the projected design produced by the slicing software. Moreover, to investigate the shape of the fabricated micropatterns, we used PDMS stamps of the PETG micropatterns. This simple method provides an indirect approach where PDMS stamps reflect the shape structure of the micropatterns. The cross-sections of the PDMS stamps indicated the formation of aligned half-cylinder-shaped micropatterns. We found that increasing the micropatterns deposition height- an equivalent to the width of half-cylinder-shaped micropattern- has proportionately led to the generations of larger micropatterns without the compromise of the shape of micropatterns where the ratio of the width to the heights was kept constant (Figure 1C). Moreover, the electron scanning microscopy (SEM) images confirmed the generation of smoothly aligned micropatterns with respect to the printing deposition heights (Figure 1D)

### 3.2. C2C12 Cell Culturing in the 3D-PMMD, Cell Orientation, and Morphology

C2C12 myoblast cells were seeded in the microchannels of 3D-PMMDs and cultured for three days. During the three-day culture in the devices, C2C12 cells were proliferated to cover the entire microchannel. To confirm cell adherence, we performed fluorescence staining of the actin fibers (F-actin). Cells’ adherence on the micropatterns was confirmed. Moreover, cells were parallelly organized on the aligned micropatterns (Figure S1).

**Figure 2:**
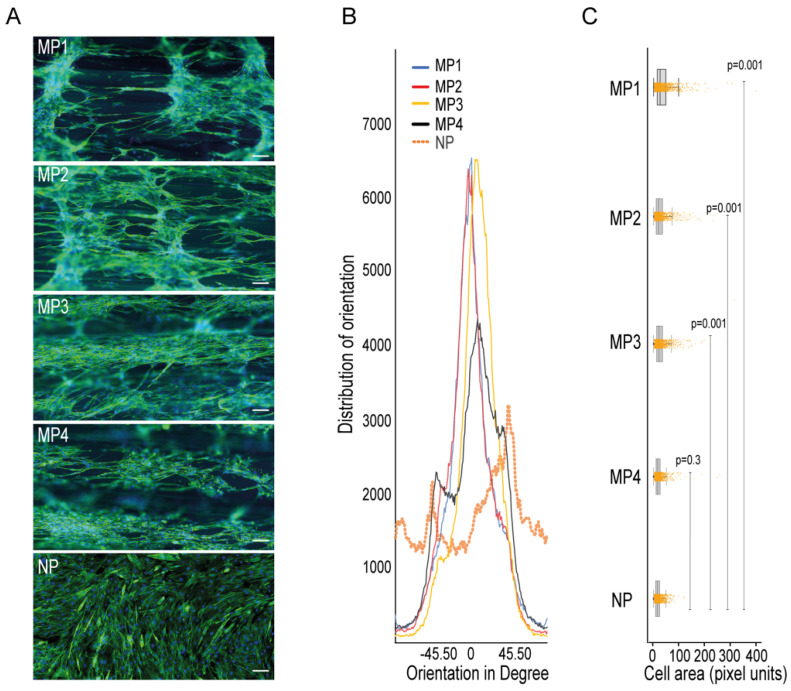
Impact of 3D-PMMD on cells orientation and morphology. (**A**) Cells were grown in microfluidic devices with different micropatterns as well as in conventional flat dishes. After the differentiation induction, cells were stained with F-actin for the analysis of cells orientation and morphology; green, F-actin; blue, DAPI; scale bar of 100 µm. (**B**) Representative histograms of cell orientation measurement at different dimensions; 100 µm (MP1), 200 µm (MP2), 400 µm (MP4), and none patterned conventional dishes (NP); distribution of orientation indicates the counts of pixel of objects based on their structure tensor. (**C**) Box plot showing the single-cell morphology analysis of cells area measurement in 3D-PMMD versus cells in conventional culturing dishes; box limits indicate the 25th and 75th percentiles. Each sample contains 1100 cells of a total random sample number of four. *P* values were obtained by Tukey’s HSD comparison test.

To check the ability of the micropatterns in 3D-PMMD in aligning myotubes, we seeded the C2C12 cells in the devices that were made with different dimensions and then investigated the cells orientations compared to cells that were grown in conventional cell culturing dishes that had no patterns. We noticed that after cell differentiation, the myotubes that were grown in the culturing dishes had a random orientation. In contrast, the myotubes that were grown on 3D-PMMD had an alignment angle in the 0° direction with respect to the dimension of the micropattern (Figure 2A,B). However, in 100 µm micropattern width, cells were also vertically aligned unlike the micropatterns at a width of 200 µm, 300 µm, and 400 µm where unidirectional orientation was prevalent. We then investigated the myotubes morphologies and we found that in myotubes that were grown on the micropatterns there was a significant increase in cells area as compared to those that were grown in the conventional culturing dishes (Figure 2C).

### 3.3. Integration with the Electrical Stimulus

**Figure 3:**
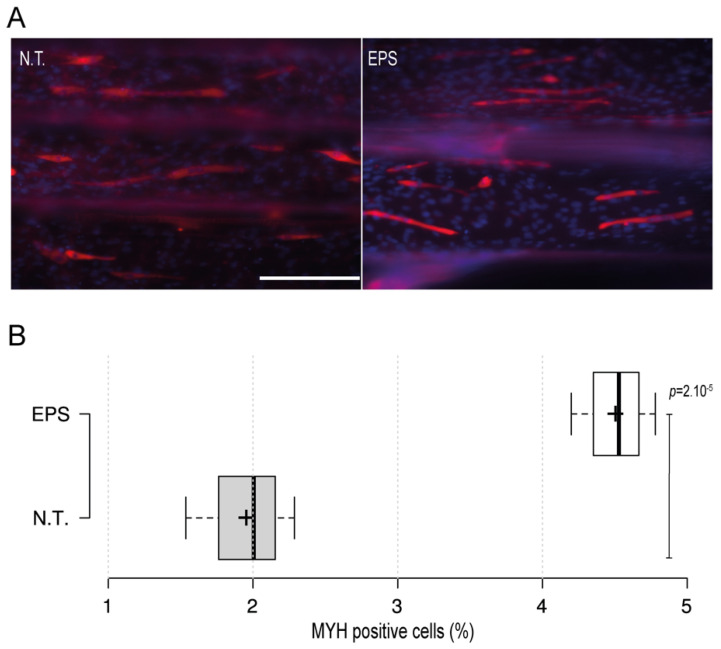
The impact of the electrical pulse stimuli (EPS) on myotubes differentiation. On day four after the initiation of the differentiation, cells were exposed to EPS for 3 h, cultured for 24 h, and then immunofluorescence staining of the myogenic MYH was conducted. (**A**) Fluorescence microscopy images of MYH expression in cells under no treatment conditions (N.T.) and EPS conditions. Red: MHY, Blue: DAPI; Scale bar of 200 µm. (**B**) Box plot showing MYH positive cells populations in 3D-PMMD under N.T and EPS conditions; box limits indicate the 25th and 75th; (*n* = 4). *p* values were obtained by unpaired *t* test.

Electrical stimulus modality is a common and important method that is usually employed with skeletal muscles to recapitulate the excitation trigger for myotubes during exercise. To further investigate the applicability of the 3D-PMMD with the electrical pulse stimulus (EPS) modality, we have designed carbon-based electrodes that can be easily positioned in the inlet and outlet areas of the device. We then applied an electric current in the frequency of 1 Hz and in intensities of 5 V or 10 V. The application of EPS in 10 V, caused cells detachment as compared with the control no treatment group (data not shown). However, by minimizing the intensity and the exposure time to 5 V for 3 h, cells were kept intact. Moreover, after the EPS exposure, cells were further cultured for 24 h and then we performed fluorescence immunostaining of the myogenic markers (MYH). Interestingly, we found that in cells that were exposed to EPS, there was a significant increase in the percentage of MYH-positive cells as compared to the control no-treatment group where there were fewer MYH-positive cells (Figure 3A,B).

## 4. Discussion

The initial prototyping of micropatterned devices is important for the development of biomimetic preclinical models of human organs, such as in skeletal muscles where the construction of aligned myotubes is critical [34,35]. However, the process of fabrication of micropatterns using conventional microfabrication methods require expertise and involve high expenses.

Here we introduce a simple FDM-based 3D printing technology for the fabrication of micropatterned microfluidic devices (3D-PMMD) made of biocompatible clear PETG polymer for the growing and expansion of skeletal muscles cells. The PETG polymer is widely used in the biomedical field as a biocompatible substance that has no toxicity concerns during cell culturing [29]. Moreover, with the employment of the clear PETG, we could achieve a fair degree of clarity and light permeability, thus enabling the visual observation of cells during the culturing process.

By the adjustment of the PETG filament deposition heights during printing, we could fabricate aligned half-cylinder-shaped micropatterns. These micropatterns were simultaneously fused during the deposition process of filaments, forming half cylinder-shaped patterns. As shown in Figure 1, we have achieved a high printing precision of half-shaped cylinders in a width and a height starting from 100 µm and 60 µm, respectively. Moreover, the increase in the dimension of the micropatterns did not affect the overall pattern shapes, which indicated that FDM printing can provide a simple yet efficient tool for the prototyping of aligned micropatterned-based microfluidic devices in high printing resolution.

We have employed a mouse C2C12 myoblast cell line that is widely used in in vitro models of the skeletal muscles [36]. As shown in Figure S1, we could observe the adherence of C2C12 myoblast cells after two days of cells culturing. Moreover, cells were aligned along the half cylinder-shaped micropatterns. Thus, these results indicated the applicability of 3D-PMMD in culturing myoblast cells, as well as the ability of 3D-PMMD in the construction of an aligned cluster of cells.

It is known that the composition and the topography of the micropattern can highly affect the myotubes orientation and morphology [35]. Therefore, we further cultured the cells in 3D-PMMD that were made with different micropatterns of a width between 100 µm to 400 µm and height between 60 µm to 160 µm, respectively. At a width of 100 µm and a height of 60 µm (MP1), although we noticed cells’ elongation and alignment, the cells tended to grow vertically rather than committing to the direction of the half cylinder-shaped pattern. On the other hand, the alignment of myotubes was noticed in micropatterns of a width between 200 µm (MP2) to 400 (MP40) compared to cells that were cultured on non-patterned flat surfaces (NP) as shown in Figure 2A,B. This indicates that the optimal alignment of myotubes can be effectively observed with micropatterns that provide enough surface area for cells elongation and inner connections during their expansion and differentiations. Therefore, our results emphasize the importance of the micropattern dimensions in 3D-PMMD for controlling myotubes alignment during the differentiation process. It is known that aligned myotubes can form fibers of a diameter range between 100 µm and 300 µm, which can lead to the formation of muscle bundles [37]. Thus, 3D-PMMD with micropatterns of a width between 200 µm (MP2) and 400 (MP4) can provide a topography that can mimic the dimension of the human fibers.

In the human body, neurons generate electrical impulses known as action potentials in frequencies between 1 and 50 Hz, which can induce muscle contractions [38]. To emulate this process in vitro, EPS in frequencies of between 1 and 10 Hz and intensities of between 1100 V are commonly employed with skeletal muscles cells [39]. To check the applicability of 3D-PMMD with EPS modality, we have employed an external carbon-based electrode that can bypass an electric pulse voltage into 3D-PMMD. We found that the application of high intensity electric current of 10 V could cause cells detachment in the microfluidic device where an elevation of temperature and the generation of reactive oxygen might have caused cells detachment. Especially that unlike, conventional culturing dishes, the cell chambers in 3D-PMMDs are much smaller, and thus temperature elevation is more likely to occur. However, by reducing the intensity to 5V, for a duration of 3 h, the cells were kept intact. Furthermore, to investigate the impact of EPS on the myogenic ability of myotubes in the early stage of differentiation (day four) in 3D-PMMD, we have checked the expression of MYH, a well know myogenic marker that is also an indicator for skeletal cells maturation [40]. It has been reported that the application of EPS can speed up the myogenic process [41]. Correspondingly, our results indicated the ability of EPS in 3D-PMMDs in enhancing the expression of MYH, thus improving the myogenic ability of cells at the early stage of differentiation.

In conclusion, 3D-PMMD has the potential for growing and aligning skeletal muscle cells in a way that mimics the physiological structure of skeletal muscles. Future studies shall highlight the applicability of 3D-PMMD in drug screening under a dynamic environment of flow application as well as under EPS during the late differentiation phase of myotubes formation.

## Data Availability

Not applicable.

## Supplementary Materials

The following are available online at https://www.mdpi.com/article/10.3390/biomimetics7010002/s1. Figure S1: C2C12 myoblast cells adherence in 3D-PMMD.

## References

[1] Bos I., Wynia K., Almansa J., Drost G., Kremer B., Kuks J. (2019). The prevalence and severity of disease-related disabilities and their impact on quality of life in neuromuscular diseases. Disabil. Rehabil..

[2] Kwan G.F., Mayosi B.M., Mocumbi A., Miranda J.J., Ezzati M., Jain Y., Robles G., Benjamin E., Subramanian S.V., Bukhman G. (2016). Endemic Cardiovascular Diseases of the Poorest Billion. Circulation.

[3] Abi-Gerges N., Miller P.E., Ghetti A. (2020). Human Heart Cardiomyocytes in Drug Discovery and Research: New Opportunities in Translational Sciences. Curr. Pharm. Biotechnol..

[4] Meißner J.D., Gros G., Scheibe R.J., Scholz M., Kubis H. (2001). Calcineurin regulates slow myosin, but not fast myosin or metabolic enzymes, during fast-to-slow transformation in rabbit skeletal muscle cell culture. J. Physiol..

[5] Mukund K., Subramaniam S. (2020). Skeletal muscle: A review of molecular structure and function, in health and disease. Wiley Interdiscip. Rev. Syst. Biol. Med..

[6] Suarez A.M.A., Brinker M.G.L., Brouwer L.A., Van Der Ham I., Harmsen M.C., Van Rijn P. (2020). Topography-Mediated Myotube and Endothelial Alignment, Differentiation, and Extracellular Matrix Organization for Skeletal Muscle Engineering. Polymers.

[7] Bakooshli M.A., Lippmann E.S., Mulcahy B., Iyer N., Nguyen C.T., Tung K., Stewart B.A., van den Dorpel H., Fuehrmann T., Shoichet M. (2019). A 3D culture model of innervated human skeletal muscle enables studies of the adult neuromuscular junction. eLife.

[8] Afshar M.E., Abraha H.Y., Bakooshli M.A., Davoudi S., Thavandiran N., Tung K., Ahn H., Ginsberg H.J., Zandstra P.W., Gilbert P.M. (2020). A 96-well culture platform enables longitudinal analyses of engineered human skeletal muscle microtissue strength. Sci. Rep..

[9] Shahin-Shamsabadi A., Selvaganapathy P.R. (2020). A 3D Self-Assembled In Vitro Model to Simulate Direct and Indirect Interactions between Adipocytes and Skeletal Muscle Cells. Adv. Biosyst..

[10] Webb R.C. (2003). Smooth muscle contraction and relaxation. Adv. Physiol. Educ..

[11] Jalili-Firoozinezhad S., Gazzaniga F.S., Calamari E.L., Camacho D., Fadel C.W., Bein A., Swenor B., Nestor B., Cronce M., Tovaglieri A. (2019). A complex human gut microbiome cultured in an anaerobic intestine-on-a-chip. Nat. Biomed. Eng..

[12] Konishi S., Fujita T., Hattori K., Kono Y., Matsushita Y. (2015). An openable artificial intestinal tract system for the in vitro evaluation of medicines. Microsyst. Nanoeng..

[13] Khetani S.R., Bhatia S.N. (2008). Microscale culture of human liver cells for drug development. Nat. Biotechnol..

[14] Huh D., Matthews B.D., Mammoto A., Montoya-Zavala M., Hsin H.Y., Ingber D.E. (2010). Reconstituting Organ-Level Lung Functions on a Chip. Science.

[15] Abdalkader R., Kamei K.-I. (2020). Multi-corneal barrier-on-a-chip to recapitulate eye blinking shear stress forces. Lab A Chip.

[16] Abdalkader R., Chaleckis R., Wheelock C.E., Kamei K.-I. (2021). Spatiotemporal determination of metabolite activities in the corneal epithelium on a chip. Exp. Eye Res..

[17] Sutterby E., Thurgood P., Baratchi S., Khoshmanesh K., Pirogova E. (2020). Microfluidic Skin-on-a-Chip Models: Toward Biomimetic Artificial Skin. Small.

[18] Akay M., Hite J., Avci N.G., Fan Y., Akay Y., Lu G., Zhu J.-J. (2018). Drug Screening of Human GBM Spheroids in Brain Cancer Chip. Sci. Rep..

[19] Voldman J., Gray M.L., Schmidt M.A. (1999). Microfabrication in Biology and Medicine. Annu. Rev. Biomed. Eng..

[20] Li N., Tourovskaia A., Folch A. (2003). Biology on a chip: Microfabrication for studying the behavior of cultured cells. Crit. Rev. Biomed. Eng..

[21] Betancourt T., Brannon-Peppas L. (2006). Micro- and nanofabrication methods in nanotechnological medical and pharmaceutical devices. Int. J. Nanomed..

[22] Chen Y., Rousseaux F., Haghiri-Gosnet A., Kupka R., Ravet M., Simon G., Launois H. (1996). Proximity X-ray lithography as a quick replication technique in nanofabrication: Recent progress and perspectives. Microelectron. Eng..

[23] Decher G. (1997). Fuzzy Nanoassemblies: Toward Layered Polymeric Multicomposites. Science.

[24] Schäffer E., Thurn-Albrecht T., Russell T.P., Steiner U. (2000). Electrically induced structure formation and pattern transfer. Nature.

[25] Duong L.H., Chen P.-C. (2019). Simple and low-cost production of hybrid 3D-printed microfluidic devices. Biomicrofluidics.

[26] Chen C., Mehl B.T., Munshi A.S., Townsend A.D., Spence D.M., Martin R.S. (2016). 3D-printed microfluidic devices: Fabrication, advantages and limitations—A mini review. Anal. Methods.

[27] Szykiedans K., Credo W. (2016). Mechanical Properties of FDM and SLA Low-cost 3-D Prints. Procedia Eng..

[28] Katschnig M., Wallner J., Janics T., Burgstaller C., Zemann W., Holzer C. (2020). Biofunctional Glycol-Modified Polyethylene Terephthalate and Thermoplastic Polyurethane Implants by Extrusion-Based Additive Manufacturing for Medical 3D Maxillofacial Defect Reconstruction. Polymers.

[29] Hassan M.H., Omar A.M., Daskalakis E., Hou Y., Huang B., Strashnov I., Grieve B.D., Bártolo P. (2020). The Potential of Polyethylene Terephthalate Glycol as Biomaterial for Bone Tissue Engineering. Polymers.

[30] Chung J.J., Im H., Kim S.H., Park J.W., Jung Y. (2020). Toward Biomimetic Scaffolds for Tissue Engineering: 3D Printing Techniques in Regenerative Medicine. Front. Bioeng. Biotechnol..

[31] Carvalho V., Gonçalves I., Lage T., Rodrigues R., Minas G., Teixeira S., Moita A., Hori T., Kaji H., Lima R. (2021). 3D Printing Techniques and Their Applications to Organ-on-a-Chip Platforms: A Systematic Review. Sensors.

[32] McQuin C., Goodman A., Chernyshev V., Kamentsky L., Cimini B.A., Karhohs K.W., Doan M., Ding L., Rafelski S.M., Thirstrup D. (2018). CellProfiler 3.0: Next-generation image processing for biology. PLoS Biol..

[33] (2020). Renesh Bedre Bioinformatics data analysis and visualization toolkit. Zenodo.

[34] Denes L.T., Riley L.A., Mijares J.R., Arboleda J.D., McKee K., Esser K.A., Wang E.T. (2019). Culturing C2C12 myotubes on micromolded gelatin hydrogels accelerates myotube maturation. Skelet. Muscle.

[35] Bettadapur A., Suh G.C., Geisse N.A., Wang E.R., Hua C., Huber H.A., Viscio A.A., Kim J.Y., Strickland J.B., McCain M.L. (2016). Prolonged Culture of Aligned Skeletal Myotubes on Micromolded Gelatin Hydrogels. Sci. Rep..

[36] Katagiri T., Yamaguchi A., Komaki M., Abe E., Takahashi N., Ikeda T., Rosen V., Wozney J.M., Fujisawa-Sehara A., Suda T. (1994). Bone morphogenetic protein-2 converts the differentiation pathway of C2C12 myoblasts into the osteoblast lineage. J. Cell Biol..

[37] Almonacid Suarez A.M., Zhou Q., Van Rijn P., Harmsen M.C. (2019). Directional topography gradients drive optimum alignment and differentiation of human myoblasts. J. Tissue Eng. Regen. Med..

[38] Hopkins P.M. (2006). Skeletal muscle physiology. Contin. Educ. Anaesth. Crit. Care Pain.

[39] Konishi S., Hashimoto T., Nakabuchi T., Ozeki T., Kajita H. (2021). Cell and tissue system capable of automated culture, stimulation, and monitor with the aim of feedback control of organs-on-a-chip. Sci. Rep..

[40] Agarwal M., Sharma A., Kumar P., Kumar A., Bharadwaj A., Saini M., Kardon G., Mathew S.J. (2020). Myosin heavy chain-embryonic regulates skeletal muscle differentiation during mammalian development. Development.

[41] Gogh I.J.E.-V., Alex S., Stienstra R., Brenkman A.B., Kersten S., Kalkhoven E. (2015). Electric Pulse Stimulation of Myotubes as an In Vitro Exercise Model: Cell-Mediated and Non-Cell-Mediated Effects. Sci. Rep..

