# Supplementary material for: The Development of Biomimetic Aligned Skeletal Muscles in a Fully 3D Printed Microfluidic Device
Source: Biomimetics (Basel). 2021 Dec 21;7(1):2. doi: 10.3390/biomimetics7010002 (PMC8788470; doi:10.3390/biomimetics7010002)
Supplement: Supplementary file 1 [file biomimetics-07-00002-s001.zip › biomimetics-1516219-supplementary.pdf]

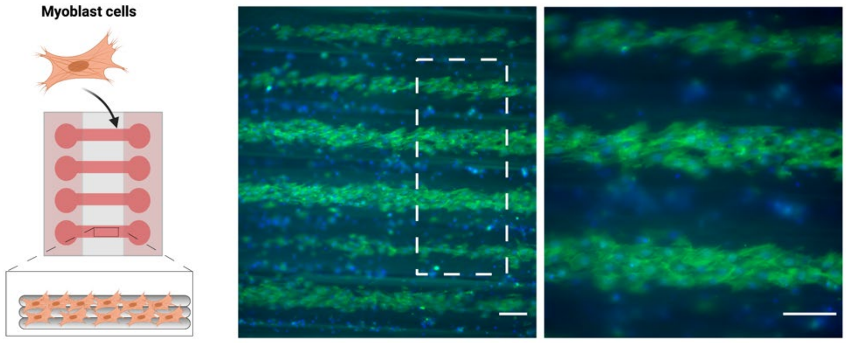

Figure S1. C2C12 myoblast cells adherence in 3D-PMMD. Cells were cultured on 3D-PMMD with a micropattern dimension of 200μm (MP2) for three days. Then, the cells' adherence and structure were analyzed by the fluorescence staining of F-actin. Green, F-actin; blue, DAPI; scale bar of 200 μm.
